# Dose-dependent social-cognitive effects of intranasal oxytocin delivered with novel Breath Powered device in adults with autism spectrum disorder: a randomized placebo-controlled double-blind crossover trial

**DOI:** 10.1038/tp.2017.103

**Published:** 2017-05-23

**Authors:** D S Quintana, L T Westlye, S Hope, T Nærland, T Elvsåshagen, E Dørum, Ø Rustan, M Valstad, L Rezvaya, H Lishaugen, E Stensønes, S Yaqub, K T Smerud, R A Mahmoud, P G Djupesland, O A Andreassen

**Affiliations:** 1 NORMENT, KG Jebsen Centre for Psychosis Research, Institute of Clinical Medicine, University of Oslo, Oslo, Norway; 2 Division of Mental Health and Addiction, Oslo University Hospital, Oslo, Norway; 3 Department of Psychology, University of Oslo, Oslo, Norway; 4 Department of Neuro Habilitation, Oslo University Hospital, Oslo, Norway; 5 NevSom, Department of Rare Disorders and Disabilities, Oslo University Hospital, Oslo, Norway; 6 Department of Neurology, Oslo University Hospital, Oslo, Norway; 7 Institute of Clinical Medicine, University of Oslo, Oslo, Norway; 8 Smerud Medical Research International AS, Oslo, Norway; 9 OptiNose US Inc, Yardley, PA, USA; 10 OptiNose AS, Oslo, Norway

## Abstract

The neuropeptide oxytocin has shown promise as a treatment for symptoms of autism spectrum disorders (ASD). However, clinical research progress has been hampered by a poor understanding of oxytocin’s dose–response and sub-optimal intranasal delivery methods. We examined two doses of oxytocin delivered using a novel Breath Powered intranasal delivery device designed to improve direct nose-to-brain activity in a double-blind, crossover, randomized, placebo-controlled trial. In a randomized sequence of single-dose sessions, 17 male adults with ASD received 8 international units (IU) oxytocin, 24IU oxytocin or placebo followed by four social-cognitive tasks. We observed an omnibus main effect of treatment on the primary outcome measure of overt emotion salience as measured by emotional ratings of faces (*η*^2^=0.18). Compared to placebo, 8IU treatment increased overt emotion salience (*P*=0.02, *d*=0.63). There was no statistically significant increase after 24IU treatment (*P*=0.12, *d*=0.4). The effects after 8IU oxytocin were observed despite no significant increase in peripheral blood plasma oxytocin concentrations. We found no significant effects for reading the mind in the eyes task performance or secondary outcome social-cognitive tasks (emotional dot probe and face-morphing). To our knowledge, this is the first trial to assess the dose-dependent effects of a single oxytocin administration in autism, with results indicating that a low dose of oxytocin can significantly modulate overt emotion salience despite minimal systemic exposure.

## Introduction

Autism spectrum disorder (ASD) is a group of neurodevelopmental disorders characterized by persistent impairments in social interaction and communication, and restricted patterns of interests and behaviors.^1^ Although ASD is a diverse disorder with a variety of presentations, deficits in social behavior are unifying features of ASD that encompass the most commonly reported symptoms that continue throughout adolescence and adulthood.^2^ Social interaction problems can have a considerable impact on quality of life with many individuals with ASD reporting impairments in relationships and reciprocal conversation ability,^2, 3^ which contribute to poorer outcomes in education, employment, community inclusion and independent living.^4, 5^ Despite a high prevalence of up to 1 in 88 individuals,^6^ current non-pharmacological therapy options for ASD are either resource intensive (20–40 h of behavioral intervention per week) or have low-to-moderate effectiveness.^7^ Pharmacotherapy options for ASD symptoms are also limited. Many drugs are used in an attempt to address different downstream aspects of the disease, such as melatonin for sleep difficulties.^8^ However, there are with no approved treatments available to address the core feature of social dysfunction, with commonly used medications only targeting the core feature of repetitive behaviors with considerable side-effects (for example, risperidone and aripiprazole^9^). Considering these factors, there is an urgent need for new pharmacotherapies to improve the core symptom of social dysfunction in ASD.

Intranasal oxytocin has emerged as a potential treatment addressing social dysfunction. The importance of the oxytocin system in mammalian social behaviors including social memory, recognition and attachment has been established using a variety of animal models since 1980s.^10, 11, 12, 13^ Buoyed by this pre-clinical animal evidence together with reports that oxytocin increases trust^14^ and modulates neural circuitry for social cognition in humans,^15^ there was a marked increase in human oxytocin research from the mid-to-late 2000s. A succession of studies reported modest improvements in social cognition after use of nasal oxytocin spray in both neurotypical^16, 17, 18^ and psychiatric^19, 20, 21^ populations, stoking intense scientific and lay interest.^22, 23^ However, a series of negative results,^24, 25, 26^ mixed meta-analytic results^27^ and doubts surrounding oxytocin research methods^28^ somewhat dampened initial enthusiasm. Given these findings, researchers have begun to recalibrate their expectations of oxytocin by more detailed investigation of its social-cognitive effects. For instance, the notion that oxytocin is exclusively a pro-social hormone has been largely abandoned,^29^ with converging evidence suggesting that oxytocin increases social salience^30^ and approach-related behaviors,^31^ regardless of valence.

In parallel with efforts to better understand contextual and individual factors that moderate oxytocin response,^30^ recent research has focused on how the mechanisms used for nasal drug administration may influence oxytocin’s effects, potentially complicating clinical study results and slowing the progress of translational research with intranasal oxytocin for treatment of psychiatric conditions.^32, 33^ First, the means of transport of oxytocin from the nasal cavity to the brain is not well understood.^34^ Although it is assumed that oxytocin travels via olfactory and trigeminal nerves to the brain rather than across the blood–brain barrier via circulating blood, this has not been directly tested in humans. Second, the optimal oxytocin dose for humans is unknown. The majority of ASD oxytocin trials in older adolescents and adults evaluated 24 international units (IU) of nasal spray oxytocin,^35^ yet there is no justification for selecting this dose beyond precedence, despite pre-clinical research demonstrating that lower doses may be more efficacious.^36, 37^ Third, currently used pump-actuated conventional nasal spray devices for intranasal oxytocin administration are not optimal for the delivery of molecules to the brain.^32^ Only a small fraction of liquid delivered with such devices reaches the upper-posterior region of the nasal cavity,^38^ which is innervated by the olfactory and trigeminal nerve fibers that are believed to be key targets for nose-to-brain delivery.^22^ Addressing these administration obstacles, we recently reported a study in neurotypical adult males comparing response to an emotion sensitivity task after a single dose of either intranasal oxytocin 8IU or 24IU, intravenous oxytocin (1IU), or placebo in a double-dummy and double-blind crossover trial using a novel Breath Powered device shown to enhance deposition in intranasal sites targeted for nose-to-brain transport.^38, 39^ A single administration of intranasal 8IU oxytocin-modulated social cognition, whereas intravenous administration did not, despite comparable peripheral blood concentrations of oxytocin after intranasal and intravenous oxytocin administration.^40^ This indicates that nose-to-brain transport, rather than transport across the blood–brain barrier via peripherally circulating oxytocin, influences social cognition. Moreover, neural evidence from the same data set also suggested that intranasal 8IU (but not the other oxytocin treatments) reduced amygdala activation during the presentation of social stimuli.^41^

Another barrier to the translation of pre-clinical oxytocin research relates to the study design issues. Many oxytocin studies are statistically underpowered, with clinical studies having an average of 12% statistical power to detect the median Cohen’s *d* of 0.32.^28^ Therefore, the corresponding average false-negative rate is large (88%), which probably contributes to poor replication rates. Although statistical power can be improved by increasing sample sizes—a common refrain in oxytocin trial report discussion sections—a more efficient and sensible approach is to boost effect sizes. For instance, effect sizes in oxytocin studies can be increased by carefully optimizing administration methods and experimental design. This is an especially attractive alternative for clinical trials, as recruiting and testing 79 individuals with ASD in a within-participants trial—the number of participants needed to achieve 80% power for the small-to-medium average effect size of *d*=0.32—would be difficult for many research groups considering that 17 is the median sample size of single-dose crossover oxytocin studies in ASD.^35^

Research has yet to investigate the dose-dependent response to intranasal oxytocin in ASD using optimized intranasal delivery. This randomized, double-blind, 3-way crossover trial in volunteers with ASD compared social-cognitive response between three treatments delivered via a Breath Powered delivery system (OptiNose, Oslo, Norway): ‘low dose’ (8IU) oxytocin, ‘higher dose’ (24IU) oxytocin and placebo. Social cognition is a complex suite of processes that cannot be encapsulated by a single experimental task. Thus, we employed four tasks detailed below to measure different elements of social cognition: theory of mind, emotional salience of overt stimuli, emotional salience of covert stimuli and the speed of recognizing overt emotional stimuli. As ASD is associated with deficits in identifying both positive and negative emotions, the social salience tasks examined the perception of both happiness and anger in social stimuli.^42^

## Materials and methods

### Participants

Participants were recruited through advertisements distributed via the Autism Society of Norway to user networks and specialist clinicians in the Oslo area. Eligible participants were male, aged 18 to 35 (inclusive), and had received a diagnosis of ASD from a specialized pediatric or psychiatric institution via multidisciplinary teams. Written confirmation of ICD-10 criteria ASD diagnosis was obtained from their treating clinicians and quality-controlled by a specialist study psychiatrist. Only males were selected because of their overrepresentation in ASD^1^ and to achieve a more homogenous study population. Exclusion criteria included psychiatric co-morbidity requiring acute intervention (for example, psychosis spectrum disorders) or IQ<75. A screening visit occurred before randomization at Oslo University Hospital. The Wechsler Abbreviated Scale of Intelligence^43^ and the Mini-International Neuropsychiatric Interview^44^ were administered by trained graduate students under the supervision of study physicians and clinical psychologists to index IQ and confirm the absence of psychiatric illnesses requiring intervention, respectively. A physical examination was performed by study physicians and nurses, including a 12-lead ECG and routine blood samples. Study physicians also confirmed normal nasal anatomy and patency in participants via physical examination under the supervision of an otolaryngologist, consistent with recent recommendations.^32^ Acoustic rhinometry data were collected by trained study staff under otolaryngologist supervision (SRE 2000; RhinoMetrics, Lynge, Denmark), yielding nasal valve dimensions (minimum cross-sectional area, summed left and right dimensions) and nasal cavity volume 2–5 cm from the nostrils. This trial was approved by the Regional Committee for Medical and Health Research Ethics (REC South East) and participants provided written informed consent before they participated. The study is registered at the EU Clinical Trials register (EudraCT no.: 2014-005452-26).

### Study design

**Figure 1:**
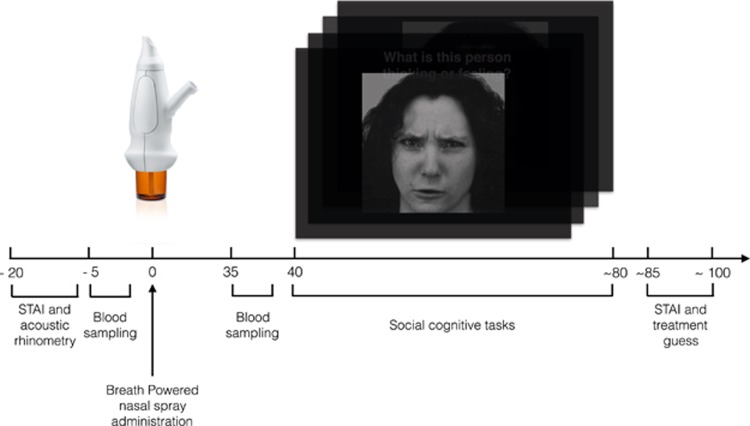
Task design. Participants were administered an intranasal solution with the social-cognitive tasks beginning 40 min after intranasal administration. Blood samples and the STAI responses (state-trait anxiety questionnaire) were also collected twice, before and after intranasal administration. Time is shown in minutes.

Participants received 8IU oxytocin intranasally, 24IU oxytocin intranasally, and an intranasal placebo treatment in a randomized, placebo-controlled, double-blind, three-period crossover design. Participants were randomized to one of six treatment sequences, using a three-period, three-treatment Latin square method (ABC—ACB—BAC—BCA—CAB—CBA in a 1:1:1 ratio; Supplementary Figure S1), with a minimum of 24 h between treatments to ensure adequate washout. Three participants, who were later randomized for treatment, took part in pilot tests of the social-cognitive and nasal spray administration procedures with open label placebo solution. An independent statistician (Smerud Medical Research International, Oslo, Norway) provided the randomization code, and both the participants and the research team were blinded to treatment. A pharmaceutical service provider (Farma Holding, Oslo, Norway) filled the oxytocin and placebo (matching liquid vehicle) formulations into the Breath Powered devices. All participants completed practice administration at every treatment session using an empty Breath Powered device under the supervision of study staff, before self-administering an intranasal treatment using another device. Participants began the social-cognitive tasks (see details below) 40 min after treatment administration in the following order during every visit: emotion sensitivity, Reading the Mind in the Eyes Test (RMET), emotional dot probe and emotional face-morphing (Figure 1). Each task took ~10 min to complete. Analysis of prior study data derived using the same emotion sensitivity primary outcome measure and Breath Powered device^40^ revealed a large effect size (partial *η*^2^=0.14). A power analysis using G*Power software^45^ indicated that a sample size of 18 would achieve 90% power for a repeated measures design, given a large effect size (partial *η*^2^=0.14) and *α*=0.05.

### Breath Powered delivery device for nasal spray administration

The breath powered, closed-palate, bi-directional nasal spray (‘Breath Powered’) device (also known as the Exhalation Delivery System) capitalizes on two aspects of nasal anatomy to facilitate efficient posterior and superior delivery of medication in the nasal cavity^46^ (Supplementary Figure S2). As the user blows through the mouth against a resistance the soft palate automatically closes, creating an airtight seal isolating the nasal cavity from the oral cavity, preventing lung deposition and limiting gastrointestinal deposition.^47^ An optimized sealing nosepiece helps direct the exhaled breath and oxytocin aerosol into the upper-posterior nasal cavity. With a closed soft palate, airflow enters via one nostril and deposits the drug aerosol on target sites and then exits from the other nostril (that is, bi-directional delivery). These conditions create a positive variable pressure in the nasal cavity, which balances pressure across the soft palate, to prevent over-elevation and ensure a patent communication around and behind the nasal septum while also expanding the nasal valve and narrow slit-like nasal passages. This mechanism has been shown to produce improved delivery of drug beyond the nasal valve to target regions in the upper and posterior nasal cavity.^46^ As in a previous study, the device was further optimized for nose-to-brain deliver with an elongated nosepiece and sideways flexible tip to improve delivery to the most upper and posterior segments of the nasal cavity.^40, 48^

### Co-primary outcome measures

We had two primary outcome measures: an overt emotion sensitivity task previously demonstrated to be modulated by oxytocin delivered via the Breath Powered device^40^ and a Norwegian translation of the RMET^49^ a commonly used task in oxytocin research^17, 19, 26, 50, 51^ to assess theory of mind performance. We hypothesized that oxytocin would increase emotion sensitivity and salience, and improve RMET performance. For the emotion sensitivity task, participants were presented with 20 male and female faces as used previously;^40, 52^ displaying angry, happy and emotionally ambiguous facial expressions derived from the Karolinska Directed Emotional Faces database.^53^ The task consisted of five blocks with 20 trials in each block. Each trial of ~6–8 s duration comprised the following sequence: fixation cross of 2 s duration→face presentation of 1 s duration→Q1 of 10 s duration (maximum response window, which terminated after participant response). Participants were asked either: ‘How angry is this person?’ (anchors: not angry—very angry) or ‘How happy is this person?’ (anchors: not happy—very happy). Participants were asked to rank their answer on a numerical rating scale from 1 to 5, with initial location of the cursor on the numerical rating scale randomized for each question. The primary outcome measures were the mean ratings for each category. The RMET is a 36-item battery indexing theory of mind ability,^49^ whereby participants are shown eye region images and asked which of four possible descriptions best describes what the person in the images is thinking or feeling. The percentage of correct responses was used as the outcome measure, as per prior research.^19^

### Secondary outcome measures

Secondary outcomes included performance on an emotional dot probe task^54^ (covert emotional salience) and an emotional face-morphing task (speed of recognizing overt emotional stimuli). On the basis of prior reports, we hypothesized that oxytocin would increase covert emotional salience in the dot probe task^55, 56^ and increase the speed of recognizing overt emotional stimuli in the face-morphing task.^57, 58^ The dot probe task assesses attentional preference or bias between two stimuli that are presented for a short period of time. Each trial began with a fixation cross for 100 ms, followed by a face stimuli pair (using the same stimuli set from the emotion sensitivity task). One of three pairs of stimuli were presented: angry-ambiguous, happy-ambiguous and ambiguous-ambiguous. The stimuli (40 faces) were drawn from the same stimulus set as the emotion sensitivity task. Pairs were presented for 500 ms, with the probe appearing directly after the faces were present in the place of one of the faces. The probe either appeared behind the target (congruent trial), which was the emotional face for the angry-ambiguous and happy-ambiguous pairs, or non-target (incongruent trial). Participants were asked to indicate where the probe appeared as fast as possible by pressing one of two keys. A total of 160 trials were presented. To create a measure of attentional bias (ms), which was the outcome measure for this task for each stimuli pair (angry and happy), reaction times from congruent trials were subtracted from incongruent trials.

A face-morphing task was developed using the same stimuli to assess speed of emotion recognition. Faces morphed from ambiguous faces to either happy (50%) or angry faces (50%) over 10 s. Morphing videos were created using Fantamorph software (version 5.4.2, Abrosoft, USA). When selecting the starting frame as the ambiguous face and the end frame as the emotional face, each video displays a consistent 10-s morph between these two faces. Participants did not see the same face twice. Participants were instructed to indicate when they could recognize the emotion as either happy or angry. There were 38 trials in total.

The forty-item state-trait anxiety inventory STAI^59^ was also administered before intranasal administration to index state and trait anxiety. The twenty-item state anxiety portion of the STAI was administered again after the completion of the social cognition tasks to assess changes in state anxiety. After completing the STAI, participants were asked to guess which treatment they were randomized to for the present experimental session (oxytocin or placebo).

### Pharmacokinetics

Blood samples were collected to assess peripheral levels of oxytocin, arginine vasopressin (AVP), and cortisol at baseline and 40 min after administration of study medication. Blood samples were centrifuged at 4 °C within 5 min of blood draw, after which plasma was frozen at −80 °C. Oxytocin concentrations were assessed with enzyme-linked immunosorbent assay (ELISA) using commercially available kits (Enzo Life Sciences, Farmingdale, NY, USA). AVP concentration was assessed with competitive radioimmunoassay using commercially available kits (BÜHLMANN Laboratories, Schönenbuch, Switzerland). Cortisol concentrations were measured by luminescence immunoassay (Siemens Immulite 2000XPi, Erlangen, Germany). The Oslo University Hospital hormone lab performed all assays using standard techniques (including sample extraction).

### Statistical analysis

Statistical analysis was conducted using the R statistical package^60^ and JASP (https://jasp-stats.org) to examine the impact of treatment on social-cognitive and pharmacokinetic outcomes. A multilevel linear mixed-model (LMM) approach using the ‘nlme’ package (http://CRAN.R-project.org/package=nlme) was adopted to assess the effect of treatment on social-cognitive outcomes measures. This approach compares the fit of a null LMM model against a main effect LMM model, which yields a likelihood ratio and *P-*value. LMMs were chosen as repeated-measure analyses to assess main effects because they do not rely on complete data sets or the assumption of compound symmetry. For any significant main effects (*P*<0.05), *post-hoc* tests were performed to compare each treatment condition (with Tukey adjustment of critical *P*-values to correct for multiple comparisons). Cohen’s *d* was calculated as a measure of effect size with values of 0.2, 0.5 and 0.8 interpreted as small, medium and large effect sizes, respectively.^61^ For comparison and the calculation of effect sizes, repeated measures ANOVAs were also performed if there was <10% missing data, with Huynh-Feldt corrected statistics presented if sphericity was violated. Eta-squared (*η*^2^) was computed in JASP as a measure of ANOVA effect size, with values of 0.01, 0.06 and 0.14 interpreted as small, medium and large effect sizes, respectively.^61^ Figures illustrating main effects and interactions contain SE bars corrected for within-subjects data.^62^ This approach helps to ensure that non-overlapping SE bars better represent significant group mean differences, as uncorrected SE bars can be misleading when visualizing within-subjects data.^63^

## Results

Twenty-six volunteers were assessed for eligibility, and seventeen participants aged 19–35 years (*M*=24.76, s.d.=4.75) were randomized (Supplementary Figure S1; mean full-scale IQ=109.8 (SD=12.1); mean body mass index=26.7 (SD=6.1)). Nine participants were excluded either because they declined to participate further (*n*=2), had a full-scale IQ<75 (*n*=2), fulfilled criteria for a psychosis spectrum disorder (*n*=2), displayed an abnormal 12-lead electrocardiogram, (*n*=2), or had clinically significant blood test results (*n*=1). Five eligible participants fulfilled criteria for a co-morbid psychiatric disorder (agoraphobia (*n*=2), major depressive disorder (*n*=2), obsessive compulsive disorder (*n*=1)). On average, 13 days (s.d.=16) elapsed between each treatment session (range: 1–72). Every randomized participant completed all three treatments. Recruitment commenced April 2015 and all treatments were completed by February 2016.

### Primary outcome measures

**Figure 2:**
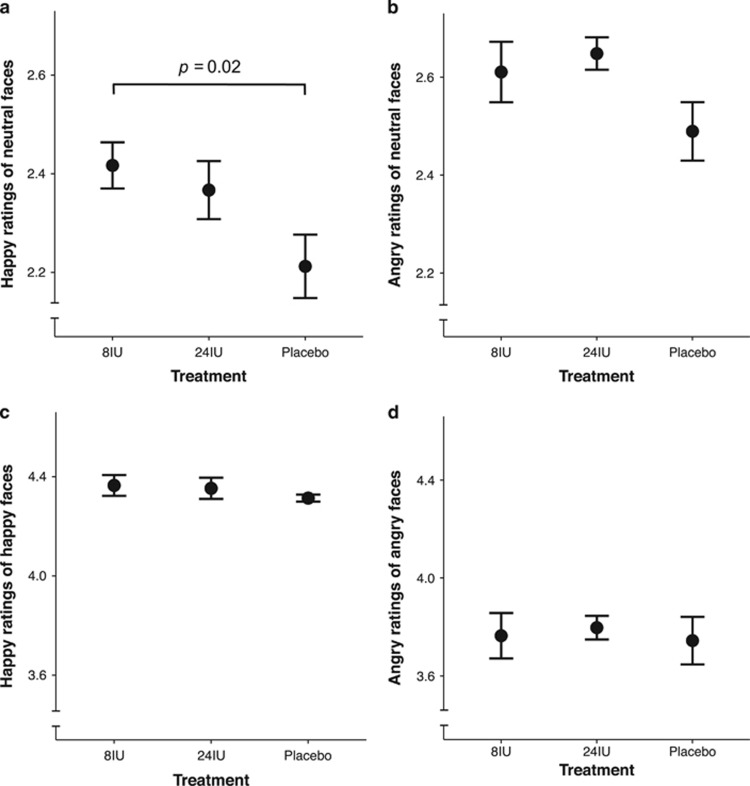
Mean scores for emotion sensitivity ratings. Happy ratings of ambiguous faces were increased after 8 international units (IU) oxytocin treatment (**a**). Angry ratings of ambiguous faces were also increased after 8IU treatment; however, this was on the border of statistical significance (**b**). There were no significant differences in happy ratings of happy faces (**c**) or angry ratings of angry faces (**d**). Error bars represent standard error of the mean and were corrected for a within-subject design.^62, 63^

**Figure 3:**
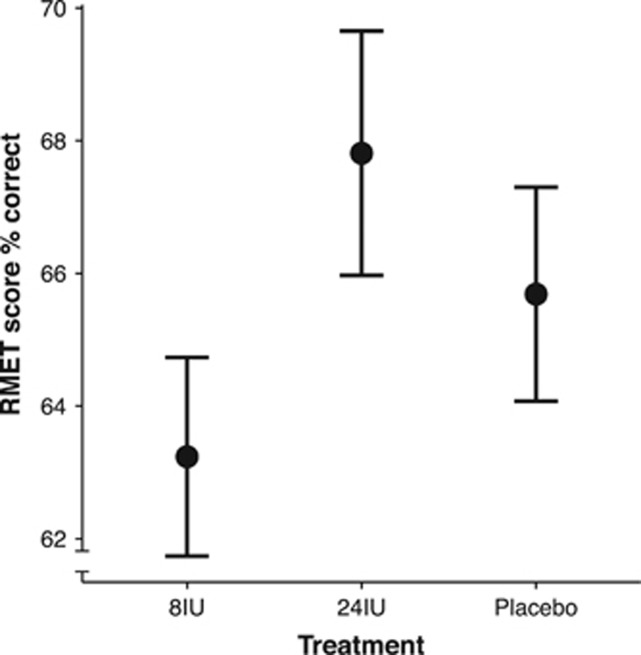
Mean RMET scores. There was also no significant difference in Reading the Mind in the Eyes Test (RMET) performance between treatment conditions. Error bars represent standard error of the mean and were corrected for a within-subject design.^62, 63^ IU, international units.

Treatment condition had a statistically significant effect on the perception of happiness in ambiguous faces, *χ*^2^(2)=6.71, *P*=0.03 (Figure 2a; Table 1). *Post-hoc* tests with Tukey-adjusted *P*-values for multiple comparisons revealed that ratings of happiness after 8IU oxytocin were significantly higher than ratings after placebo (*P*=0.02), a difference associated with a medium-to-large effect size (*d*=0.63). There was no significant difference in the perception of happiness between 24IU and placebo (*P*=0.12, *d*=0.4) or 8IU and 24IU (*P*=0.8; *d*=0.18). For comparison, a repeated measures ANOVA revealed a similar outcome to the LMM with a significant main effect (F_(2, 32)_=3.49, *P*=0.04), which was associated with a large effect size (*η*^2^=0.18). There was also evidence of a main effect of treatment on the perception of anger in ambiguous faces, but this was on the border of statistical significance, *χ*^2^(2)=4.83, *P*=0.09 (Figure 2b). Tukey-adjusted multiple comparisons revealed that the increase in anger ratings after 24IU compared to placebo treatments was also on the border of statistical significance (*P*=0.07, *d*=0.64). A repeated measures ANOVA with Huynh-Feldt sphericity correction provided equivalent statistics to the LMM (F_(1,56, 24.97)_=2.44, *P*=0.1), which was associated with a medium-to-large effect size (*η*^2^=0.13). There was no significant effect of treatment on the happy ratings of happy faces (*χ*^2^(2)=1.19, *P*=0.55; Figure 2c) or angry ratings of angry faces (*χ*^2^(2)=0.22, *P*=0.89; Figure 2d). There was also no significant effect of treatment (*χ*^2^(2)=3.83, *P*=0.15; Figure 3) or visit order (that is, practice effects; *χ*^2^(2)=1.79, *P*=0.41) on RMET performance.

### Secondary outcome measures

There was no statistically significant main effect of treatment condition on speed or accuracy of detection for happy (speed: *χ*^2^(2)=0.02, *P*=0.99; accuracy: *χ*^2^(2)=0.43, *P*=0.8) or angry (speed: *χ*^2^(2)=0.23, *P*=0.89; accuracy: *χ*^2^(2)=5.39, *P*=0.07) face morphs (Supplementary Table S1). Treatment effects on speed of identifying faces for correct trials only were similar for happy (74% of all trials; *χ*^2^(2)=2.8, *P*=0.25) and angry faces (70% of all trials; *χ*^2^(2)=0.18, *P*=0.91). Results from the dot probe task also revealed there was no main effect of treatment on attentional bias towards happy (*χ*^2^(2)=2.48, *P*=0.29) or angry (*χ*^2^(2)=0.76, *P*=0.68) faces (Supplementary Table S1).

### Pharmacokinetics, safety and anxiety measures

**Figure 4:**
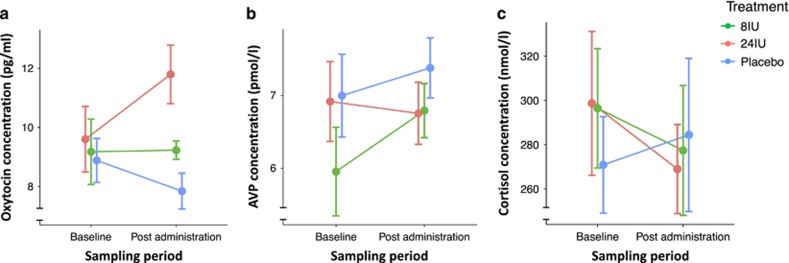
Hormone pharmacokinetics. Pharmacokinetics of plasma oxytocin (**a**), arginine vasopressin (AVP) (**b**), and cortisol (**c**) before and after each treatment administration. Error bars represent standard error of the mean and were corrected for a within-subjects design.^62, 63^ IU, international units.

The main effect of treatment condition on peripheral oxytocin concentrations was on the border of statistical significance (*χ*^2^(2)=5.48, *P*=0.06; Figure 4a). There was no main effect of time (*χ*^2^(1)=0.3, *P*=0.58), however, the treatment × time interaction effect was on the border of statistical significance (*χ*^2^(2)=5.37, *P*=0.07). Simple main effects revealed no treatment condition effect at baseline (*χ*^2^(2)=0.17, *P*=0.92); however, there was a treatment condition, simple main effect on peripheral oxytocin concentrations post treatment administration (*χ*^2^(2)=13.79, *P*=0.001). *Post-hoc* comparisons (Tukey-adjusted) revealed increased peripheral oxytocin concentrations after 24IU compared to 8IU (*P*=0.01) and placebo (*P*<0.001). There was no difference in peripheral oxytocin concentration after 8IU and placebo (*P*=0.45). Regarding peripheral AVP concentrations (Figure 4b), there was no main effect of treatment condition (*χ*^2^(2)=2.03, *P*=0.36), time (*χ*^2^(1)=1.89, *P*=0.17), or treatment × time interaction (*χ*^2^(2)=4.2, *P*=0.12). There was also no main effect of treatment (*χ*^2^(2)=0.24, *P*=0.89), time (*χ*^2^(1)=1.65, *P*=0.2), or treatment × time interaction (*χ*^2^(2)=0.34, *P*=0.84) for peripheral cortisol concentrations (Figure 4c).

There was no significant main effect of treatment condition on state anxiety, as measured by the STAI-state questionnaire, (*χ*^2^(2)=0.65, *P*=0.72) but a significant time effect (*χ*^2^(1)=17.45, *P*<0.001) with a reduction of ratings from baseline to the conclusion of testing. However, there was no significant treatment × time interaction (*χ*^2^(2)=0.5, *P*=0.78) for state anxiety. There was also no effect of treatment group on nasal valve dimensions (*χ*^2^(2)=2.65, *P*=0.27), or the total volume of the nasal cavity 2–5 cm deep from the nostrils (*χ*^2^(2)=1.23, *P*=0.54). There was also no statistically significant relationship between nasal valve dimensions or total volume of the nasal cavity (2–5 cm deep from the nostrils) and performance on any of the social cognition tasks (Supplementary Tables S2 and S3). A *χ*^2^ test revealed no difference between treatment conditions on whether participants could correctly identify the treatment they were administered (*χ*^2^ (2)=2.57, *P*=0.28). Adverse events reported after treatment administrations were transient and easily tolerated (for example, dizziness associated with blood collection, fatigue and headache), and were distributed across the three-treatment arms (8IU, 4 reports; 24IU, 4 reports; and placebo, 2 reports).

## Discussion

This pre-registered randomized controlled trial was designed to assess the dose-dependent effects of intranasal oxytocin for social-cognitive performance in ASD. Here, we show that a single 8IU intranasal administration of oxytocin significantly increases the overt emotional salience of happiness in ambiguous faces compared to placebo, providing additional support that oxytocin treatment may help ameliorate a core feature of ASD. Although there was no significant difference in mean rating of happiness between 8IU and 24IU oxytocin, only scores after 8IU were significantly higher than ratings after placebo treatment. There was also a main effect of treatment condition on the perception of anger associated with a medium-to-large effect size; however, this was on the border of statistical significance, making conclusions less clear. Exploratory *post-hoc* analysis, albeit non-significant, suggested that anger ratings were increased after 24IU oxytocin treatment compared to placebo, a difference coupled with a medium effect size. There were no statistically significant main effects on the RMET primary outcome measure, or secondary outcome measures (dot probe and face-morphing). Altogether, these results suggest that oxytocin administration may influence the sensitivity of overt emotion perception, particularly happiness, in ambiguous facial stimuli in adults with ASD, but might not influence the speed of emotion recognition, implicit bias towards emotional stimuli, or theory of mind performance.

Individuals with ASD are less sensitive to both positively and negatively valenced social information.^42, 64, 65^ Consequently, potential treatments addressing social dysfunction in ASD need to address the whole gamut of social cues—not just positive cues. In the present trial, we observed an increase in the salience of both happiness and anger after intranasal oxytocin, although only the increased perception of happiness after an 8IU oxytocin dose reached statistical significance (whereas the increased perception of anger was only on the border of statistical significance despite a medium-to-large effect size). The data is somewhat consistent with the social salience hypothesis,^66^ which suggests that oxytocin modulates the salience of both positive and negative social cues. For reference, compared to happiness rating of ambiguous faces in the present sample (Table 1), neurotypical adult males that completed the same task after placebo (*n*=16) had an average happiness rating of ambiguous faces of 2.51 (s.d.=0.13).^40^ Comparing these two outcomes provides tentative evidence that oxytocin treatment in adult ASD may facilitate a shift towards neurotypical responses to social stimuli, at least for positive stimuli. Although we previously observed a reduction in angry ratings of ambiguous faces after 8IU administration,^40^ this was found in a sample of neurotypical adults tested in a unique fMRI environment.

Prior research using a similar sample size (*n*=16) demonstrated that intranasal oxytocin improves RMET performance in male adolescents with ASD;^19^ however, we did not observe an effect on RMET performance in the present older sample of adult males with ASD. This may have been because of ceiling effects on performance due to the sampling of a population with less severe ASD. For comparison, after placebo administration the present sample scored 65.7% correct on the RMET, whereas previous research examining the impact of oxytocin in an ASD population reported scores of approximately 45% correct RMET items.^19^ The present score after placebo administration is also slightly higher than ASD norms for RMET performance (61%).^49^ In addition, we did not find any statistically main effects for performance on the secondary outcome dot probe or face-morphing measures. This suggests that exogenous oxytocin might not influence the emotional salience of covert stimuli or the speed of recognizing overt emotional stimuli in young male adults with ASD. An alternative explanation is that the timing of these two tasks (~60–80 min post-administration) may have missed peak central concentrations of oxytocin, which is thought to be around 35–50 min post-administration. Indeed, previous effects on the face-morphing or the dot probe tasks occurred at when these tasks were performed during this time period.^55, 56, 57, 58^ Finally, the study sample was appropriately powered to detect a main effect for the overt emotion salience tasks, however, it may not have had enough power to detect differences in these secondary tasks.

Animal research has shown the potential for long-term adverse effects after large oxytocin doses.^67, 68^ Although only minimal adverse events have been reported in oxytocin ASD trials to date,^69^ it is still crucial to determine the lowest efficacious dose to limit adverse events and exposure to widely distributed peripheral oxytocin receptors.^70^ There were no significant differences in peripheral oxytocin concentrations after 8IU and placebo treatments. This suggests that 8 IU oxytocin delivered with the Breath Powered device increased emotion sensitivity without significantly elevating peripheral oxytocin, which supports the possibility of direct nose-to-brain transport with this delivery mechanism and potential to achieve therapeutic benefit while minimizing adverse effects due to peripheral oxytocin receptor activation. There was also no difference in AVP levels, suggesting no peripheral cross-reactivity, which is more likely to occur with higher levels of oxytocin exposure.^71^ Research is increasingly pointing towards lower oxytocin doses being more (or at least equivalently) efficacious than higher doses.^36, 37, 72, 73^ The present result also adds to existing behavioral^40^ and neural^41^ evidence that lower oxytocin doses delivered with the Breath Powered device may be more efficacious, at least for single administration trials. Alternatively, 24IU oxytocin delivered via traditional nasal devices may offer equivalent central levels because of substantial loss of the delivered drug. Delivery of the drug to the non-ciliated mucosa anterior to the nasal valve,^39^ may drip out of the nostril or be sniffed along the floor of the nose and swallowed. As traditional nasal spray devices deliver less liquid to nose-to-brain targets in the upper-posterior region of the nasal cavity, this could limit the fraction reaching the brain and central activity. Future research is needed to directly compare performance of the Breath Powered device and traditional device for delivering intranasal oxytocin. Regardless, even if 24IU oxytocin delivered with traditional device provides equivalent central activity as 8IU delivered with the Breath Powered device, the latter device appears to reduce peripheral exposure to oxytocin.

The study had some limitations worth noting. First, the participant sample was a population of young adult males without intellectual disability. Future work in broader populations is needed to assess the generalizability to a wider ASD population. Despite fulfilling criteria for ASD, a measure of disorder severity was not collected. However, as a main effect on a primary outcome was observed, without any need for subtyping participants on any demographic or clinical variable, this suggests that the effect is robust across a relatively heterogeneous male ASD population with a range of verbal, non-verbal, and general cognitive function aptitudes (WASI full-scale IQ mean: 109.8, s.d.: 12.1, range: 89–138). Second, although our sample size matched the median sample size for oxytocin crossover studies,^35^ this still may be considered as small. However, a power analysis based on effect sizes using the same primary outcome measure in healthy controls^40^ revealed a sample of 18 was sufficient to achieve statistical power of 90%, which is exceptionally higher than the 12% average statistical power of oxytocin clinical trials to detect the mean effect size across trials.^28^

Altogether, our study provides initial evidence that a low dose of oxytocin delivered with the Breath Powered device modulates overt social salience in ASD. To our knowledge, this is the first demonstration of such an effect in a heterogeneous adult male ASD population. Moreover, this social-cognitive effect of oxytocin administration was achieved with minimal systemic exposure. Future work will be required to determine the effect of multiple 8IU oxytocin dosing with Breath Powered device on social interaction measures and quality of life in people with ASD.

## Figures and Tables

**Table 1:** Primary social cognition outcome measures

	*8IU OT*	*24IU OT*	*Placebo*	*Pairwise comparison* P*-values*		
				*8IU vs placebo*	*24IU vs placebo*	*8IU vs 24IU*
*Emotion sensitivity*						
Happy ratings of neutral faces	2.42 (0.05)	2.37 (0.06)	2.21 (0.06)	0.02**	0.12	0.8
Angry ratings of neutral faces	2.61 (0.06)	2.65 (0.03)	2.49 (0.06)	0.22	0.07*	0.86
Happy ratings of happy faces	3.76 (0.09)	3.8 (0.05)	3.74 (0.1)	0.54	0.7	0.97
Angry ratings of angry faces	−0.25 (0.21)	−0.15 (0.25)	0.53 (0.22)	0.98	0.89	0.95
						
*RMET*						
Percentage correct	63.24 (1.5)	67.81 (1.84)	65.69 (1.61)	0.53	0.62	0.11

Abbreviations: IU, international units; RMET, Reading the Mind in the Eyes Test. **P<0.05.

*P<0.01.

Values represent means with standard error in parenthesis.

## References

[bib1] American Psychiatric AssociationDiagnostic and Statistical Manual of Mental Disorders (DSM-5®). Americann Psychiatric Association: Washington, DC, 2013.

[bib2] Shattuck PT, Seltzer MM, Greenberg JS, Orsmond GI, Bolt D, Kring S et al. Change in autism symptoms and maladaptive behaviors in adolescents and adults with an autism spectrum disorder. J Autism Dev Disord 2007; 37: 1735–1747. 17146700 10.1007/s10803-006-0307-7 PMC3265360

[bib3] Orsmond GI, Shattuck PT, Cooper BP, Sterzing PR, Anderson KA. Social participation among young adults with an autism spectrum disorder. J Autism Dev Disord 2013; 43: 2710–2719. 23615687 10.1007/s10803-013-1833-8 PMC3795788

[bib4] Howlin P, Moss P, Savage S, Rutter M. Social outcomes in mid-to later adulthood among individuals diagnosed with autism and average nonverbal IQ as children. J Am Acad Child Adolesc Psychiatry 2013; 52: 572–581. 23702446 10.1016/j.jaac.2013.02.017

[bib5] Gray KM, Keating CM, Taffe JR, Brereton AV, Einfeld SL, Reardon TC et al. Adult outcomes in autism: community inclusion and living skills. J Autism Dev Disord 2014; 44: 3006–3015. 24915930 10.1007/s10803-014-2159-x

[bib6] Baio J. Prevalence of autism spectrum disorders: autism and developmental disabilities monitoring network, 14 Sites, United States, 2008. Morbidity and Mortality Weekly Report. Surveillance Summaries. Volume 61(3). Centers for Disease Control and Prevention, 2012. 22456193

[bib7] Lai M-C, Lombardo MV, Baron-Cohen S. Autism. Lancet 383: 896–910. 24074734 10.1016/S0140-6736(13)61539-1

[bib8] Rossignol DA, Frye RE. Melatonin in autism spectrum disorders: a systematic review and meta‐analysis. Dev Med Child Neurol 2011; 53: 783–792. 21518346 10.1111/j.1469-8749.2011.03980.x

[bib9] McPheeters ML, Warren Z, Sathe N, Bruzek JL, Krishnaswami S, Jerome RN et al. A systematic review of medical treatments for children with autism spectrum disorders. Pediatrics 2011; 127: e1312–e1321. 21464191 10.1542/peds.2011-0427

[bib10] Pedersen CA, Ascher JA, Monroe YL, Prange AJ. Oxytocin induces maternal behavior in virgin female rats. Science 1982; 216: 648–650. 7071605 10.1126/science.7071605

[bib11] Fahrbach S, Morrell J, Pfaff D. Possible role for endogenous oxytocin in estrogen-facilitated maternal behavior in rats. Neuroendocrinology 1985; 40: 526–532. 4010891 10.1159/000124125

[bib12] Winslow JT, Insel TR. The social deficits of the oxytocin knockout mouse. Neuropeptides 2002; 36: 221–229. 12359512 10.1054/npep.2002.0909

[bib13] Insel TR, Shapiro LE. Oxytocin receptor distribution reflects social organization in monogamous and polygamous voles. Proc Natl Acad Sci 1992; 89: 5981–5985. 1321430 10.1073/pnas.89.13.5981 PMC402122

[bib14] Kosfeld M, Heinrichs M, Zak PJ, Fischbacher U, Fehr E. Oxytocin increases trust in humans. Nature 2005; 435: 673–676. 15931222 10.1038/nature03701

[bib15] Kirsch P, Esslinger C, Chen Q, Mier D, Lis S, Siddhanti S et al. Oxytocin modulates neural circuitry for social cognition and fear in humans. J Neurosci 2005; 25: 11489–11493. 16339042 10.1523/JNEUROSCI.3984-05.2005 PMC6725903

[bib16] Guastella AJ, Mitchell PB, Dadds MR. Oxytocin increases gaze to the eye region of human faces. Biol Psychiatry 2008; 63: 3–5. 17888410 10.1016/j.biopsych.2007.06.026

[bib17] Domes G, Heinrichs M, Michel A, Berger C, Herpertz SC. Oxytocin Improves “Mind-Reading” in Humans. Biol Psychiatry 2007; 61: 731–733. 17137561 10.1016/j.biopsych.2006.07.015

[bib18] Guastella AJ, Mitchell PB, Mathews F. Oxytocin enhances the encoding of positive social memories in humans. Biol Psychiatry 2008; 64: 256–258. 18343353 10.1016/j.biopsych.2008.02.008

[bib19] Guastella AJ, Einfeld SL, Gray KM, Rinehart NJ, Tonge BJ, Lambert TJ et al. Intranasal oxytocin improves emotion recognition for youth with autism spectrum disorders. Biol Psychiatry 2010; 67: 692–694. 19897177 10.1016/j.biopsych.2009.09.020

[bib20] Andari E, Duhamel J-R, Zalla T, Herbrecht E, Leboyer M, Sirigu A. Promoting social behavior with oxytocin in high-functioning autism spectrum disorders. PNAS 2010; 107: 4389–4394. 20160081 10.1073/pnas.0910249107 PMC2840168

[bib21] Feifel D, Macdonald K, Nguyen A, Cobb P, Warlan H, Galangue B et al. Adjunctive intranasal oxytocin reduces symptoms in schizophrenia patients. Biol Psychiatry 2010; 68: 678–680. 20615494 10.1016/j.biopsych.2010.04.039

[bib22] Quintana DS, Guastella AJ, Westlye LT, Andreassen OA. The promise and pitfalls of intranasally administering psychopharmacological agents for the treatment of psychiatric disorders. Mol Psychiatry 2016; 21: 29–38. 26552590 10.1038/mp.2015.166

[bib23] Shamay-Tsoory S, Young LJ. Understanding the oxytocin system and its relevance to psychiatry. Biol Psychiatry 2016; 79: 150. 26723107 10.1016/j.biopsych.2015.10.014 PMC4718566

[bib24] Dadds MR, MacDonald E, Cauchi A, Williams K, Levy F, Brennan J. Nasal oxytocin for social deficits in childhood autism: a randomized controlled trial. J Autism Dev Disord 2014; 44: 521–531. 23888359 10.1007/s10803-013-1899-3

[bib25] Davis MC, Green MF, Lee J, Horan WP, Senturk D, Clarke AD et al. Oxytocin-augmented social cognitive skills training in schizophrenia. Neuropsychopharmacology 2014; 39: 2070–2077. 24637803 10.1038/npp.2014.68 PMC4104336

[bib26] Cacciotti-Saija C, Langdon R, Ward PB, Hickie IB, Scott E, Naismith SL et al. A double-blind randomized controlled trial of oxytocin nasal spray and social cognition training for young people with early psychosis. Schizophr Bull 2015; 41: 483–493. 24962607 10.1093/schbul/sbu094 PMC4332939

[bib27] Bakermans-Kranenburg M, Van Ijzendoorn M. Sniffing around oxytocin: review and meta-analyses of trials in healthy and clinical groups with implications for pharmacotherapy. Transl Psychiatry 2013; 3: e258. 23695233 10.1038/tp.2013.34 PMC3669921

[bib28] Walum H, Waldman ID, Young LJ. Statistical and methodological considerations for the interpretation of intranasal oxytocin studies. Biol Psychiatry 2016; 79: 251–257. 26210057 10.1016/j.biopsych.2015.06.016 PMC4690817

[bib29] Nave G, Camerer C, McCullough M. Does oxytocin increase trust in humans? A critical review of research. Perspect Psychol Sci 2015; 10: 772–789. 26581735 10.1177/1745691615600138

[bib30] Bartz JA, Zaki J, Bolger N, Ochsner KN. Social effects of oxytocin in humans: context and person matter. Trends Cogn Sci 2011; 15: 301–309. 21696997 10.1016/j.tics.2011.05.002

[bib31] Kemp AH, Guastella AJ. The role of oxytocin in human affect a novel hypothesis. Curr Dir Psychol Sci 2011; 20: 222–231.

[bib32] Guastella AJ, Hickie IB, McGuinness MM, Otis M, Woods EA, Disinger HM et al. Recommendations for the standardisation of oxytocin nasal administration and guidelines for its reporting in human research. Psychoneuroendocrinology 2013; 38: 612–625. 23265311 10.1016/j.psyneuen.2012.11.019

[bib33] Quintana DS, Alvares GA, Hickie IB, Guastella AJ. Do delivery routes of intranasally administered oxytocin account for observed effects on social cognition and behavior? A two-level model. Neurosci Biobehav Rev 2015; 49: 182–192. 25526824 10.1016/j.neubiorev.2014.12.011

[bib34] Leng G, Ludwig M. Intranasal oxytocin: myths and delusions. Biol Psychiatry 2016; 79: 243–250. 26049207 10.1016/j.biopsych.2015.05.003

[bib35] Alvares GA, Quintana DS, Whitehouse AJ. Beyond the hype and hope: critical considerations for intranasal oxytocin research in autism spectrum disorder. Autism Res 2016; 10: 25–41. 27651096 10.1002/aur.1692

[bib36] Benelli A, Bertolini A, Poggioli R, Menozzi B, Basaglia R, Arletti R. Polymodal dose–response curve for oxytocin in the social recognition test. Neuropeptides 1995; 28: 251–255. 7596490 10.1016/0143-4179(95)90029-2

[bib37] Popik P, Vetulani J, Van Ree JM. Low doses of oxytocin facilitate social recognition in rats. Psychopharmacology 1992; 106: 71–74. 1738795 10.1007/BF02253591

[bib38] Djupesland PG, Skretting A, Winderen M, Holand T. Breath actuated device improves delivery to target sites beyond the nasal valve. Laryngoscope 2006; 116: 466–472. 16540911 10.1097/01.MLG.0000199741.08517.99

[bib39] Djupesland PG, Skretting A. Nasal deposition and clearance in man: comparison of a bidirectional powder device and a traditional liquid spray pump. J Aerosol Med Pulm Drug Deliv 2012; 25: 280–289. 22251061 10.1089/jamp.2011.0924

[bib40] Quintana DS, Westlye LT, Rustan ØG, Tesli N, Poppy CL, Smevik H et al. Low dose oxytocin delivered intranasally with Breath Powered device affects social-cognitive behavior: a randomized 4-way crossover trial with nasal cavity dimension assessment. Transl Psychiatry 2015; 5: 1–9. 10.1038/tp.2015.93 PMC5068727 26171983

[bib41] Quintana DS, Westlye LT, Alnæs D, Rustan Ø, Kaufmann T, Smerud K et al. Low dose intranasal oxytocin delivered with Breath Powered device dampens amygdala response to emotional stimuli: a peripheral effect-controlled within-subjects randomized dose-response fMRI trial. Psychoneuroendocrinology 2016; 69: 180–188. 27107209 10.1016/j.psyneuen.2016.04.010

[bib42] Wright B, Clarke N, Jordan J, Young AW, Clarke P, Miles J et al. Emotion recognition in faces and the use of visual context Vo in young people with high-functioning autism spectrum disorders. Autism 2008; 12: 607–626. 19005031 10.1177/1362361308097118

[bib43] Wechsler D. Weschsler Abbreviated Scale of Intelligence. Psychological Corporation: San Antonio, TX, 1999.

[bib44] Lecrubier Y, Sheehan D, Weiller E, Amorim P, Bonora I, Harnett Sheehan K et al. The Mini International Neuropsychiatric Interview (MINI). A short diagnostic structured interview: reliability and validity according to the CIDI. Eur Psychiatry 1997; 12: 224–231.

[bib45] Faul F, Erdfelder E, Lang A-G, Buchner A. G* Power 3: a flexible statistical power analysis program for the social, behavioral, and biomedical sciences. Behav Res Methods 2007; 39: 175–191. 17695343 10.3758/bf03193146

[bib46] Djupesland PG. Nasal drug delivery devices: characteristics and performance in a clinical perspective—a review. Drug Deliv Transl Res 2012; 3: 42–62. 23316447 10.1007/s13346-012-0108-9 PMC3539067

[bib47] Djupesland PG, Messina JC, Mahmoud RA. The nasal approach to delivering treatment for brain diseases: an anatomic, physiologic, and delivery technology overview. Ther Deliv 2014; 5: 709–733. 25090283 10.4155/tde.14.41

[bib48] Djupesland PG, Mahmoud RA, Messina JC. Accessing the brain: the nose may know the way. J Cereb Blood Flow Metab 2013; 33: 793–794. 23486291 10.1038/jcbfm.2013.41 PMC3652706

[bib49] Baron-Cohen S, Wheelwright S, Hill J, Raste Y, Plumb I. The "Reading the Mind in the Eyes" Test revised version: a study with normal adults, and adults with Asperger syndrome or high-functioning autism. J Child Psychol Psychiatry 2001; 42: 241–251. 11280420

[bib50] Radke S, de Bruijn ER. Does oxytocin affect mind-reading? A replication study. Psychoneuroendocrinology 2015; 60: 75–81. 26142239 10.1016/j.psyneuen.2015.06.006

[bib51] Anagnostou E, Soorya L, Chaplin W, Bartz J, Halpern D, Wasserman S et al. Intranasal oxytocin versus placebo in the treatment of adults with autism spectrum disorders: a randomized controlled trial. Mol Autism 2012; 3: 16. 23216716 10.1186/2040-2392-3-16 PMC3539865

[bib52] Leknes S, Wessberg J, Ellingsen DM, Chelnokova O, Olausson H, Laeng B. Oxytocin enhances pupil dilation and sensitivity to 'hidden' emotional expressions. Soc Cogn Affect Neurosci 2012; 8: 741–749. 22648957 10.1093/scan/nss062 PMC3791062

[bib53] Lundqvist D, Flykt A, Öhman A. The Karolinska directed emotional faces (KDEF). CD ROM from Department of Clinical Neuroscience, Psychology section, Karolinska Institutet, 1998, pp 91–630.

[bib54] MacLeod C, Mathews A, Tata P. Attentional bias in emotional disorders. J Abnorm Psychol 1986; 95: 15. 3700842 10.1037//0021-843x.95.1.15

[bib55] Domes G, Sibold M, Schulze L, Lischke A, Herpertz S, Heinrichs M. Intranasal oxytocin increases covert attention to positive social cues. Psychol Med 2013; 43: 1747–1753. 23146328 10.1017/S0033291712002565

[bib56] Clark-Elford R, Nathan PJ, Auyeung B, Mogg K, Bradley BP, Sule A et al. Effects of oxytocin on attention to emotional faces in healthy volunteers and highly socially anxious males. Int J Neuropsychopharmacol 2014; 18: pyu012. 25552432 10.1093/ijnp/pyu012 PMC4368883

[bib57] Marsh AA, Henry HY, Pine DS, Blair R. Oxytocin improves specific recognition of positive facial expressions. Psychopharmacology 2010; 209: 225–232. 20186397 10.1007/s00213-010-1780-4

[bib58] Averbeck B, Bobin T, Evans S, Shergill S. Emotion recognition and oxytocin in patients with schizophrenia. Psychol Med 2012; 42: 259–266. 21835090 10.1017/S0033291711001413 PMC3250086

[bib59] Spielberger CD. Manual for the State-Trait Anxiety Inventory STAI (Form Y)("Self-Evaluation Questionnaire"). Consulting Psychologists Press: Palo Alto, CA, 1983.

[bib60] R Development Core TeamR: A Language and Environment for Statistical Computing. R Foundation for Statistical Computing: Vienna, Austria, 2014.

[bib61] Cohen J. Statistical Power Analysis for the Behavioural Sciences. Lawrence Earlbaum Associates: Mahwah, NJ, 1988.

[bib62] Morey RD. Confidence intervals from normalized data: a correction to Cousineau (2005). Tutor Quant Methods Psychol 2008; 4: 61–64.

[bib63] Baguley T. Calculating and graphing within-subject confidence intervals for ANOVA. Behav Res Methods 2012; 44: 158–175. 21858605 10.3758/s13428-011-0123-7

[bib64] Wallace GL, Case LK, Harms MB, Silvers JA, Kenworthy L, Martin A. Diminished sensitivity to sad facial expressions in high functioning autism spectrum disorders is associated with symptomatology and adaptive functioning. J Autism Dev Disord 2011; 41: 1475–1486. 21347615 10.1007/s10803-010-1170-0 PMC3448486

[bib65] Kennedy DP, Adolphs R. Perception of emotions from facial expressions in high-functioning adults with autism. Neuropsychologia 2012; 50: 3313–3319. 23022433 10.1016/j.neuropsychologia.2012.09.038 PMC3518664

[bib66] Shamay-Tsoory SG, Abu-Akel A. The social salience hypothesis of oxytocin. Biol Psychiatry 2016; 79: 194–202. 26321019 10.1016/j.biopsych.2015.07.020

[bib67] Bales KL, Perkeybile AM, Conley OG, Lee MH, Guoynes CD, Downing GM et al. Chronic intranasal oxytocin causes long-term impairments in partner preference formation in male prairie voles. Biol Psychiatry 2013; 74: 180–188. 23079235 10.1016/j.biopsych.2012.08.025 PMC3556198

[bib68] Bales KL, van Westerhuyzen JA, Lewis-Reese AD, Grotte ND, Lanter JA, Carter CS. Oxytocin has dose-dependent developmental effects on pair-bonding and alloparental care in female prairie voles. Horm Behav 2007; 52: 274–279. 17553502 10.1016/j.yhbeh.2007.05.004 PMC1978481

[bib69] Ooi Y, Weng S, Kossowsky J, Gerger H, Sung M. Oxytocin and autism spectrum disorders: a systematic review and meta-analysis of randomized controlled trials. Pharmacopsychiatry 2016; 50: 5–13. 27574858 10.1055/s-0042-109400

[bib70] Gimpl G, Fahrenholz F. The oxytocin receptor system: structure, function, and regulation. Physiol Rev 2001; 81: 629–683. 11274341 10.1152/physrev.2001.81.2.629

[bib71] Manning M, Misicka A, Olma A, Bankowski K, Stoev S, Chini B et al. Oxytocin and vasopressin agonists and antagonists as research tools and potential therapeutics. J Neuroendocrinol 2012; 24: 609–628. 22375852 10.1111/j.1365-2826.2012.02303.x PMC3490377

[bib72] Hall SS, Lightbody AA, McCarthy BE, Parker KJ, Reiss AL. Effects of intranasal oxytocin on social anxiety in males with fragile X syndrome. Psychoneuroendocrinology 2012; 37: 509–518. 21862226 10.1016/j.psyneuen.2011.07.020 PMC3353652

[bib73] Cardoso C, Ellenbogen MA, Orlando MA, Bacon SL, Joober R. Intranasal oxytocin attenuates the cortisol response to physical stress: a dose–response study. Psychoneuroendocrinology 2013; 38: 399–407. 22889586 10.1016/j.psyneuen.2012.07.013

