# Supplementary material for: Dose-dependent social-cognitive effects of intranasal oxytocin delivered with novel Breath Powered device in adults with autism spectrum disorder: a randomized placebo-controlled double-blind crossover trial
Source: Transl Psychiatry. 2017 May 23;7(5):e1136–. doi: 10.1038/tp.2017.103 (PMC5584522; doi:10.1038/tp.2017.103)
Supplement: Supplementary Information [file tp2017103x1.doc]

**Dose-dependent social-cognitive effects of intranasal oxytocin delivered with novel Breath Powered device in adults with autism spectrum disorder: A randomized placebo controlled double blind crossover trial**

***Supplementary Information***

**Supplementary figure S1. Trial CONSORT diagram.**


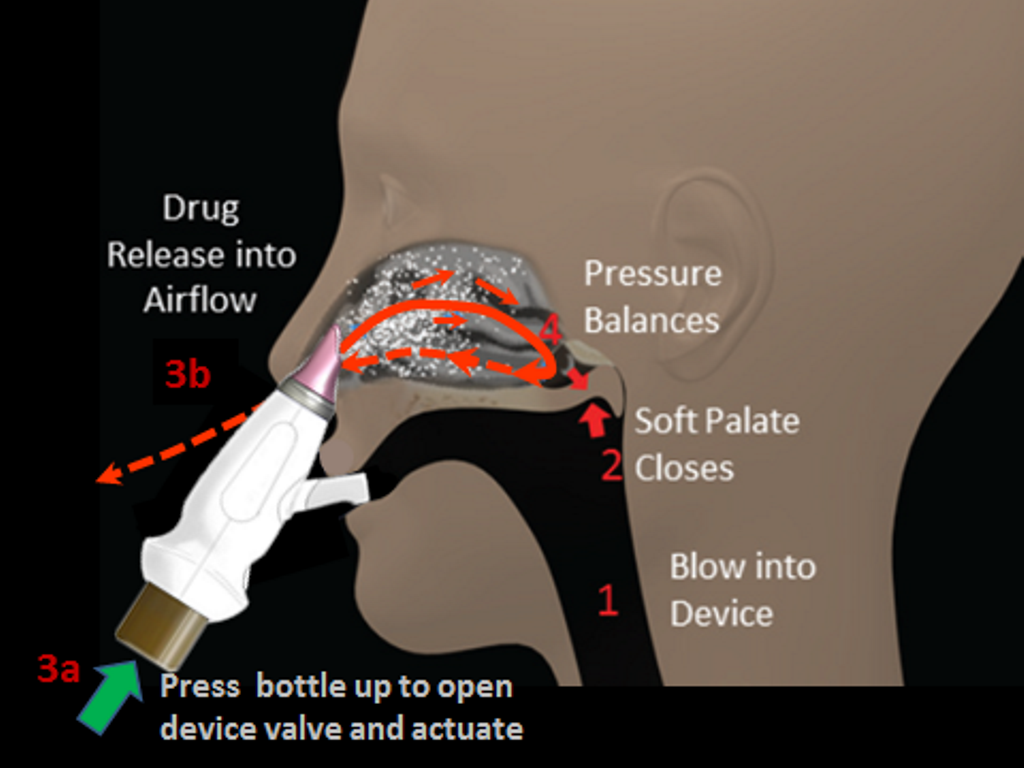


**Supplementary figure S2. The Breath Powered, closed-palate, bi-directional nasal liquid delivery device.** The user slides the optimized elongated sealing nosepiece of the device into the nostril, takes a deep breath, closes their lips around the mouthpiece and then exhales into the device (1). The intraoral pressure created by blowing into the mouthpiece closes the soft palate and creates an airtight seal (2), isolating the nasal cavity from the rest of the respiratory system. Exhaling into the device creates a positive pressure, which is released by opening of an internal valve when the user actuates the spray pump (3a). The user’s exhaled breath propels the drug to targeted nose-to-brain regions in the upper posterior nasal cavity (3b). The airflow balances pressure across the soft palate (4) enabling the exhaled breath to travel the in the opposite direction, exiting out the opposite nostril.

| **Supplementary Table S1.** Secondary social cognition outcome measures | | | | | | | |
| --- | --- | --- | --- | --- | --- | --- | --- |
|  | 8IU OT | 24IU OT | Placebo |  | Pairwise comparison p-values | | |
|  |  |  |  |  |  |  |  |
|  |  |  |  |  | 8IU vs. placebo | 24IU vs. placebo | 8IU vs. 24IU |
| Face morphing |  |  |  |  |  |  |  |
|  |  |  |  |  |  |  |  |
| Speed of recognising happy faces | 4043.8 (161.8) | 4039.3 (189.6) | 4006 (192) |  | 0.99 | 0.99 | 0.99 |
| Percentage of correct happy face trials | 97.3 (1.1) | 97.7 (0.8) | 98.2 (0.9) |  | 0.79 | 0.95 | 0.93 |
| Speed of recognising angry faces | 5262.5 (200.2) | 5183.6 (242.6) | 5350.7 (243.4) |  | 0.96 | 0.88 | 0.98 |
| Percentage of correct angry face trials | 95.33 (6.1) | 98.68 (3) | 96.8 (6.2) |  | 0.79 | 0.95 | 0.93 |
|  |  |  |  |  |  |  |  |
| Dot probe task |  |  |  |  |  |  |  |
| Attentional bias for happy faces | 0.52 (11.52) | -2.78 (11.63) | 24.92 (17.11) |  | 0.67 | 0.97 | 0.83 |
| Attentional bias for angry faces | -4.45 (8.1) | -9.19 (7.74) | -12.36 (3.5) |  | 0.32 | 0.31 | 0.98 |
| Attentional bias for neutral faces | -13.58 (8.08) | -12.05 (7.47) | -5.9 (7.67) |  | 0.53 | 0.62 | 0.11 |
|  |  |  |  |  |  |  |  |
| *Note*. Values represent means with standard error in parenthesis. | | | | | | | |
|

| **Supplementary Table S2.** The Relationship between nasal valve dimensions and social-cognitive task performance | | | | | | | |  |
| --- | --- | --- | --- | --- | --- | --- | --- | --- |
|  | 8IU OPN-OT | |  | 24IU OPN-OT | |  | Placebo | |
|  |  |  |  |  |  |  |  |  |
|  | *r* (95% CI) | *p* |  | *r* (95% CI) | *p* |  | *r* (95% CI) | *p* |
|  |  |  |  |  |  |  |  |  |
| Emotion sensitivity |  |  |  |  |  |  |  |  |
| Happy ratings of ambiguous faces | .05 (-.45, .54) | .84 |  | .08 (-.44, .55) | .78 |  | -.03 (-.5, .46) | .92 |
| Angry ratings of ambiguous faces | -.02 (-.51, .48) | .94 |  | .02 (-.48, .51) | .93 |  | -.04 (-.51, .45) | .89 |
| Happy ratings of happy faces | .3 (-.23, .69) | .26 |  | -.07 (-.54, .44) | .81 |  | -.04 (-.51, .45) | .88 |
| Angry ratings of angry faces | -.12 (-.58, .4) | .66 |  | .16 (-.37, .61) | .56 |  | .54 (.08, .81) | .02 |
|  |  |  |  |  |  |  |  |  |
| Reading the mind in the eyes task | -.1 (-.57, .41) | .7 |  | -.13 (-.6, .39) | .62 |  | -.08 (-.54, .41) | .75 |
|  |  |  |  |  |  |  |  |  |
| Emotional dot probe |  |  |  |  |  |  |  |  |
| Angry bias | .58 (.12, .84) | .02 |  | .16 (-.38, .62) | .56 |  | .53 (.07, .81) | .03 |
| Happy bias | -.04 (-.53, .46) | .87 |  | .25 (-.3, .68) | .36 |  | .07 (-.42, .53) | .78 |
|  |  |  |  |  |  |  |  |  |
| Emotional face morphing |  |  |  |  |  |  |  |  |
| Angry face identification | -.05 (-.54, .45) | .84 |  | -.19 (-.64, .36) | .5 |  | .48 (-.01, .78) | .05 |
| Happy face identification | -.04 (-.53, .47) | .88 |  | -.09 (-.58, .44) | .74 |  | .43 (-.07, .75) | .09 |
|  |  |  |  |  |  |  |  |  |
| Note: No correlations were statistically significant after Bonferroni alpha adjustment (α = .00185) | | | | | | | | |

| **Supplementary Table S3.** The Relationship between nasal cavity volume and social-cognitive task performance | | | | | | | | |
| --- | --- | --- | --- | --- | --- | --- | --- | --- |
|  | 8IU OPN-OT | |  | 24IU OPN-OT | |  | Placebo |  |
|  |  |  |  |  |  |  |  |  |
|  | *r* (95% CI) | *p* |  | *r* (95% CI) | *p* |  | *r* (95% CI) | *p* |
|  |  |  |  |  |  |  |  |  |
| Emotion sensitivity |  |  |  |  |  |  |  |  |
| Happy ratings of ambiguous faces | -.23 (-.65, .3) | .4 |  | -.34 (-.71, .19) | .2 |  | -.31 (-.69, .2) | .22 |
| Angry ratings of ambiguous faces | -.48 (-.79, .02) | .06 |  | -.24 (-.66, .29) | .37 |  | -.15 (-.59, .36) | .57 |
| Happy ratings of happy faces | -.35 (-.72, .18) | .19 |  | -.43 (-.76, .08) | .09 |  | .18 (-.33, .61) | .48 |
| Angry ratings of angry faces | -.41 (-.75, .11) | .12 |  | -.34 (-.71, .19) | .2 |  | .25 (-.26, .65) | .34 |
|  |  |  |  |  |  |  |  |  |
| Reading the mind in the eyes task | .13 (-.39, .59) | .63 |  | .16 (-.37, .61) | .56 |  | -.23 (-.28, .64) | .38 |
|  |  |  |  |  |  |  |  |  |
| Emotional dot probe |  |  |  |  |  |  |  |  |
| Angry bias | .5 (.01, .8) | .05 |  | .34 (-.21, .73) | .22 |  | .28 (-.23, .67) | .27 |
| Happy bias | -.44 (-.78, .07) | .09 |  | .3 (-.25, .7) | .28 |  | -.3 (-.68, .22) | .25 |
|  |  |  |  |  |  |  |  |  |
| Emotional face morphing |  |  |  |  |  |  |  |  |
| Angry face identification | -.12 (-.58, .4) | .65 |  | -.44 (-.78, .1) | .1 |  | -.34 (-.7, .17) | .18 |
| Happy face identification | -.22 (-.65, .31) | .41 |  | -.49 (-.8, .02) | .06 |  | -.35 (-.71, .15) | .4 |
|  |  |  |  |  |  |  |  |  |
| Note: Nasal cavity volume calculated 2 to 5 cm from the nostrils. No correlations were statistically significant after Bonferroni alpha adjustment (α = .00185). | | | | | | | | |
|  |  |  |  |  |  |  |  |  |
